# Injurious and Adulterated Beverages

**Published:** 1887-05

**Authors:** 

**Affiliations:** Montreal


### INJURIOUS AND ADULTERATED BEVERAGES.

BY AN OCCASIONAL CONTRIBUTOR, MONTREAL.

No. 2.

Dr. Carpenter, of London, England, in an address recently delivered thus forcibly describes the highly injurious effects of stimulants alike on young and old :

"Nervous stimulants have the effect of altering, even without any token of previous excess, the nutrition of the nervous system ; while thus feeling that it cannot do its work without them, there is a craving for the renewal of them. This leads me to the point upon which I would lay very great stress, and that is the duty that lies upon us all to *discourage as much as possible the early use of stimulants by children and young people.* It is during the time that the bodily organization is being formed up to the period of full growth, that everything which operates from without has the greatest potency, not merely at the time, but in forming the ultimate habit. Our fixed habits are those formed in early life, and I believe that it is simply on this account that in early life, so long as the organism is growing, the new material that is laid down, is laid down on the lines in which we exercise it, and those lines must determine what I may call the *construction of the edifice.* If that be the case in the child, the nervous system is in a state of most extraordinary activity, and it is in that period that any influence acting from without will *lay the foundation of the future mode of growth and action of the nervous system.*"

The foregoing strongly corroborates a fact well known among medical men, that the nerves act as they are *trained,* and if unduly elevated and unnaturally strained in early days, they will keep their dangerous set or tension and be more liable to be broken down in after years.

The following, selected and condensed from the *Encyclopedia Britannica,* will give some idea as to how the process of coloring tea is accomplished, and of Chinese manufacturing methods generally.

For consummate skill in the "tricks of trade" the Chinese have long been noted. "They are a self-ended people," says an old writer, "having the same reputation in Asia that the Jews have in Europe."

A century later we find Duhalde warned his readers that "the Chinese call a great many herbs by the name of tea which have no claim to that distinction."

An analysis made some years ago in England of samples, varieties of "selected pekoe," "selected caper" and "black gunpowder," proved that they were all largely adulterated, either with an imitation of tea, formed of fragments of rice or *paddy-husks* or with glazing substances, such as black lead, indigo, turmeric and an iridescent powder resembling mica. Of thirty samples of green tea, every one was found to be adulterated. The substances detected were Prussian blue, China clay, turmeric, and a white powder, variously composed, but usually consisting of kaolin, soapstone, or sulphate of lime. Five of these samples, called by the vendors gunpowder, consisted entirely of what the Chinese themselves called "*lie-tea.*" They had the candor, in this instance, to call things by their right names. This "*lie-tea*" is composed of tea dust and sand made up with rice water. Another sample was largely composed of *paddy-husks* and other fraudulent substances. Another contained a large admixture of foreign leaves. Every sample of thirty examined was artificially colored or glazed ; not a single leaf of natural green was found ; all, when deprived of their cosmetics, were either yellow, olive, brown or black.

The coloring of green tea is an operation performed exclusively for the advantage of Europeans, and the Chinese are usually quite free to exhibit the process. "Tea," they sometimes say, on such occasions, "is better without Prussian blue and gypsum, but foreigners seem to prefer a mixture of these ingredients, to give it a uniform color."

The adulterating substances are cut up into small pieces and mixed up with a paste of gum and catechu or terra japonica, reduce them to powder, color them with rose pink, and then mix them up with tea dust or black teas of inferior quality. Exhausted tea leaves mixed with a solution of gum or of terra japonica, re-dried and faced or glazed with a mixture of rose pink or black lead are used to an enormous extent. Here the fraud can best be detected by chemical analysis. Logwood is used to give a high color to the infusion of inferior black teas. If such an infusion be tested by sulphuric acid a reddish tinge will indicate its presence. Powdered talc or soapstone is used to give a deceptive bloom. The coloring and glazing used in England and America with many ingenious and recent discoveries, are of a much more injurious and poisonous kind than those used in China.

Dr. W. J. Morton, in a paper upon the above subject, read before the recent annual meeting of the American Neurological Association, held in New York, arrives at the following conclusions in regard to tea: 1st. That with it, as with any other *potent drug*, there was a proper and improper use of it. 2d. That in moderation it was a mild and pleasant stimulant followed by no harmful reaction. 3rd. Its continual and immoderate use led to a very serious group of symptoms, such as headache, vertigo, ringing in the ears, tremulousness, nervousness, exhaustion of mind and body, disinclination to mental and physical exertion, increased and irregular action of the heart and *dyspepsia*. 4th. The mental symptoms are not to be attributed to dyspepsia. 5th. It diminishes the amount of urine and retards the metamorphosis of tissue. 6th. Many of the symptoms of immoderate tea-drinking are such as might occur *without a suspicion of the real cause.*

COFFEE.

Let us now examine the question of coffee and the methods employed by ingenius dealers who supply the markets with preparations sold under this head.

Prof. L. P. Sharples, the State Assayer of Massachusetts, is making some analysis of articles of food, which are resulting in interesting discoveries. Packages of coffee have engaged his attention, but he has found very few traces of the berry itself. The following are some of the results of his examination :

Hayward & Co's French Breakfast Coffee, the label of which sets forth that only three-quarters as much of it need be used as would be required of ordinary coffee, was found to contain no coffee at all ! but to be made of green peas, burnt molssses and an "occasional grain of rye."

A package of "Pure Roasted and Ground Cape Coffee" was found to be made wholly of peas and nutshells, the latter floating when the mixture was put in water !

A package of "Kimball's First Quality Mocha and Java Coffee" contained no coffee at all, but consisted of peas and rye !

"Elinée's Extra Quality French Coffee" was almost destitute of any foreign substance, peas and rye predominating, with a few oats !

"Chase's English Breakfast Coffee" is a large consumer of peas, the traces of coffee being so slight that the assayer pronounced them accidental.

Happily these analysis have not disclosed the presence of any injurious substance, and if people, who can easily find out the cost of a pound of green coffee, expect to buy a like quantity roasted and ground for half the price, they deserve to drink weak pea soup.

To detect adulteration, the following rules are given : Take some cold water in a glass and throw upon it about half a teaspoonful of the coffee to be tested, stirring it around so as to wet the grains. Pure coffee will float and scarcely color the water. Beans and chicory sink to the bottom; chicory colors the water at once, beans more slowly. Test the part that floats by chewing it; coffee will thus be recognized by its taste; nutshells, which also float, are hard and brittle.

A species of nut which has lately come into use, strongly resembles coffee when ground up, by floating on the water as well as by its feeling between the teeth; but the difference can easily be detected because the adulterating ingredient is nearly tasteless. After subjecting the suspected article to the above test, spread some of it on a sheet of paper and examine it carefully for grains of rye, oats and peas.

The pea ingredient will frequently be found in pieces one-eighth its size and the rye in half grains; chicory is tough when taken between the teeth and has a bitter taste, different from that of coffee.

As to the roasted peas and rye which are sold instead of coffee, *it is pretty certain that they are more wholesome than the fruit of the coffee plant, being destitute of any narcotic quality;* but the thing is a fraud, and it would be better for families—cheaper at least—to roast their own peas and make their own weak pea soup.

As to chicory, it is well-known that at the restaurants in Paris and other French cities, all the coffee served to customers is largely adulterated with the roasted root of the plant, which is cultivated for that purpose, *and is much more wholesome than coffee.*

It has long been well-known that most of the nervous disorders prevalent are traceable in great measure, directly or indirectly, to the immoderate use of *tea* and coffee, nevertheless they are used extensively in almost as great quantities as ever; neualgia, sick-headache dyspepsia and a whole train of similar disorders, entailing untold suffering, might be put down to having been originated or inherited from their use in immoderate quantities.

The use of milk, in good quantity, with tea, may serve, in great degree to overcome its more injurious and powerful action, but those who drink tea without milk, will assuredly suffer very painful after-effects, either in sick-headache or some serious nervous disorder; and its continued use in this way, will surely weaken the digestion, producing dyspepsia and other kindred evils.

It may naturally be inquired, what would you propose as a substitute, if we are to give up tea? To this may be answered that there are various good wholesome and palatable substitutes for tea, as for instance, milk, cocoa, chocolate, broma, coffee, or even cold water. Any of these would be infinitely better ; they have been placed in the order as written, that their comparative value may thus be indicated ; the first *milk*, being considered best, when it can be obtained *pure* and of *good quality*. All the others are palatable and might be used according to individual taste ; coffee, however, being liable to frequent adulterations, should be used spairingly and not too strong.

Better than any of the above, however, is buttermilk ; for a summer beverage, there can be nothing more healthy and strengthening. It is excellent for weak or delicate stomachs and far better as a dinner drink than tea, coffee or water, and unlike them, does not retard but rather aids digestion.

A great physician once said that if every one knew the value of buttermilk as a drink, it would be more freely partaken of by persons who drink so excessively of other beverages, and further compared its effects upon the system, to the cleaning out of a cook stove that has been clogged up with ashes that have sifted through, filling every crevice and crack, saying that the human system is like the stove, and collects and gathers refuse matter that can in no way be exterminated from the system so effectually as by drinking buttermilk. It is also a remedy for indigestion, soothes and quiets the nerves, and is very somnolent to those who are troubled with sleeplessness. Its medicinal qualities cannot be overrated, and it should be freely used by all who can get it. Every one who values good health should drink buttermilk every day in warm weather, and let tea, coffee, and water alone. For the benefit of those who are not already aware of it, I may add, that in the churming of it, the first process of digestion is gone through, making it one of the easiest and quickest of all things to digest. It makes gastric juice, and contains properties that readily assimilate with it, with very little wear upon the digestive organs.

